# A Comparison of Owl Pellet Analysis and Conventional Trapping: A Review of Central European Cases for the Study of Small Mammal Communities and More Efficient Paleoenvironmental Reconstructions

**DOI:** 10.1002/ece3.74401

**Published:** 2026-11-10

**Authors:** Dorina Szalai, Csaba Kiss, Tamás Cserkész, Attila Virág, Attila Németh

**Affiliations:** ^1^ Department of Mineralogy and Geology University of Debrecen Debrecen Hungary; ^2^ Doctoral School of Earth Sciences University of Debrecen Debrecen Hungary; ^3^ Department of Zoology, Institute of Biology Eszterházy Károly Catholic University Eger Hungary; ^4^ Department of Zoology Hungarian Natural History Museum Budapest Hungary; ^5^ Department of Petrology and Geochemistry, Institute of Geography and Earth Sciences Eötvös Loránd University Budapest Hungary; ^6^ Department of Nature Conservation, Zoology and Game Management University of Debrecen Debrecen Hungary; ^7^ BirdLife Hungary—Hungarian Ornithological and Nature Conservation Society Budapest Hungary

**Keywords:** biodiversity monitoring, owl, owl pellet, paleoenvironment reconstruction, prey selection, sampling method, small mammal, trapping

## Abstract

Due to their rapid reproductive cycles and short generation times, small mammals—particularly rodents and shrews—are widely used as bioindicators in ecological studies. While traditional live trapping provides detailed demographic data for such monitoring, it is labour‐intensive and subject to sampling bias. In contrast, owl pellet analysis presents a non‐invasive and cost‐effective alternative for evaluating small mammal community structure over broad spatial and temporal scales, though potential prey preferences should be considered. This study evaluates the effectiveness of owl pellet analysis in comparison to conventional trapping methods for monitoring small mammal communities. We synthesised data from 26 published studies and additional unpublished field reports, comprising a uniquely robust dataset of 40 systematically processed case studies across seven countries in Central Europe, a biogeographically coherent region. In all cases, trapping and pellet collection were carried out at the same locations and during the same periods, allowing for an exceptional level of methodological consistency. Results reveal that owl pellet analysis detects higher species diversity and provides a more efficient, non‐invasive means of monitoring small mammal assemblages over larger spatial and temporal scales. However, prey preferences and hunting behaviour of owls introduce biases, often overrepresenting abundant prey such as the common vole, *Microtus arvalis* and underrepresenting species associated with specific habitats or microclimates. These biases have significant implications for biodiversity assessments and paleoenvironmental reconstructions, where accurate community composition is essential. Our findings indicate that while owl pellet analysis is a valuable tool for ecological monitoring, careful consideration of predator selectivity is necessary to accurately interpret the results. Further research into owl hunting ecology is recommended to refine the application of this method in biodiversity studies and paleoecology.

## Graphical abstract

The paper compares two commonly used methods of monitoring small mammals—owl pellet analysis and traditional trapping methods—by examining cases in Central Europe. Although owl pellet analysis detects higher species diversity and provides a more efficient, non‐invasive means of monitoring small mammal assemblages over larger spatial and temporal scales, prey preferences and hunting behaviours of owls introduce biases. These biases can have significant implications for biodiversity assessments and paleoenvironmental reconstructions, where an accurate understanding of community composition is essential.

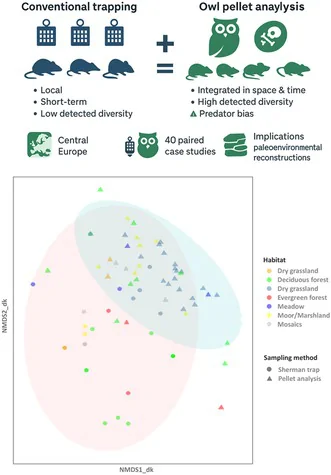

## 1 Introduction

Small mammals, particularly rodents and shrews, are widely used as indicators of environmental change because their populations respond rapidly to habitat and climatic variation. Their short life cycles and high reproductive rates make them especially informative for ecological monitoring and paleoecological reconstruction (Avenant 2005; Riegert et al. 2021; Terry 2009). Monitoring small mammal communities can provide insight into species distributions, habitat change and ecosystem integrity (Avenant 2000; Torre et al. 2016). Various direct and indirect methods are used to estimate small mammal communities, including hair traps, eDNA analysis, camera traps, owl pellet analysis and live trapping (Barja et al. 2016; Barnes and Turner 2016; Caravaggi et al. 2017; Leempoel et al. 2020; Lyet et al. 2021).

Conventional live trapping of small mammals is a labour‐intensive field method that often requires repeated sampling over multiple nights or seasons to collect representative samples of local communities (Glennon et al. 2002; Palmeirim et al. 2019). Such surveys can provide detailed demographic and morphological information, including age, sex, body mass and reproductivity (Brown and Mougeot 2011; Fuller and Roper 2010). However, detecting rare species during short sampling periods may be difficult and may require specialised survey techniques (Harkins et al. 2019). The capture probability can also vary among species depending on trap design, bait type and species‐specific behaviour (Botelho et al. 2024; O’Farrell et al. 1994; Stephens and Anderson 2014; Torre et al. 2010). Different trapping methods may also sample different components of small mammal communities, and combining several approaches may provide a more complete representation of local fauna (Mengak and Guynn Jr. 1987). Despite these challenges, live trapping remains an important tool for estimating local relative abundance and for studying population structure in small mammals. Ethical considerations may also arise in trapping studies, as capture, marking and temporary confinement can cause stress or injury if the trapping is not carefully managed (Lemckert et al. 2006; Powell and Proulx 2003).

Owl pellet analysis is widely used in many regions as it is a non‐invasive approach to monitor small mammal communities (Debus et al. 2004; Horváth et al. 2004; McDonald et al. 2014; Schoenefuss et al. 2024; Teta et al. 2010). While live trapping is essential for understanding spatio‐temporal patterns of small mammal populations (Meek et al. 2012; Riegert et al. 2021; Van Strien et al. 2015), pellet analysis can provide large faunal samples with relatively limited field effort (Charley et al. 2025). Birds of prey, particularly owls, are important predators of small mammals that swallow their prey whole and later regurgitate indigestible remains—such as bones, fur, feathers and chitin—in the form of pellets (März 2007). These remains can be identified at species level, providing information on small mammal diversity and distribution (Taylor 2003). Owls often hunt over relatively large areas and consume a wide range of small mammal prey. As a result, pellet assemblages may include species that are rarely detected in trapping surveys (Bednarz and Dinsmore 2004; Cserkész 2020; Marti and Kochert 1991). For this reason, owl pellets have been widely used in studies of small mammal diversity and community composition (McDonald et al. 2014; Oliveira et al. 2023; Schoenefuss et al. 2024; Teta et al. 2010). Pellet assemblages have also been used to investigate relationships between small mammal community composition and environmental variables such as habitat structure or climatic conditions (Glåmseter 2021; Holt et al. 2024; Romanowski and Zmihorski 2008).

An advantage of pellet analysis is that increasing sample size or geographic coverage can improve the representation of small mammal communities across the landscape without the need for capturing animals directly. However, pellet analysis also has limitations. Diet composition in owls may vary among species due to differences in body size, hunting behaviour and habitat use (Comay and Dayan 2018a; Ille 1991; Taylor 2009). In addition, predator behaviour and prey availability can influence the composition of pellet assemblages; moreover, the representation of species in pellets may differ from their relative occurrence in the surrounding environment (de la Peña et al. 2003; Torre et al. 2004).

In the Palearctic region, many pellet studies focus on the western barn owl ( *Tyto alba* ), whose diet has been widely studied in relation to small mammal communities (Andrade et al. 2016; Schoenefuss et al. 2024). Interpreting pellet assemblages in terms of small mammal abundance assumes that owl predation does not strongly bias the representation of prey species in pellets (Yom‐Tov and Wool 1997). Consistency in sampling, such as repeated pellet collection from the same roost sites, improves the comparability of pellet‐derived datasets (Terry 2010; Torre et al. 2004). Long‐term owl roosts containing large accumulations of pellets can provide valuable material for monitoring small mammal communities over a long time period (McDonald et al. 2014).

Despite these biases, owl pellet analysis is an important tool in ecology and palaeontology for investigating small mammal biodiversity and distribution across a wide range of habitats and environmental conditions. Pellet assemblages often contain numerous individuals and multiple species, providing datasets that complement information from live‐trapping studies (Heisler et al. 2016; Stefke and Landler 2020; Terry 2010).

Some studies report a positive correlation between pellet‐derived data and trapping results (Bernard et al. 2010; Terry 2010), while others report clear differences between the two methods (Torre et al. 2004). Research on small mammal communities has been conducted across a wide range of spatial scales. Many studies focus on local communities within individual reserves or limited areas (Benedek and Sirbu 2009; Drebet 2022; Horváth 1998a; Jánoska 1995; Lanszki 2004; Milchev 2015; Németh 2014; Váczi et al. 2019). Other studies compile large datasets or examine patterns across broader geographic regions (Allen et al. 2019; Escribano‐Compains et al. 2016; Heisler et al. 2016; Romano et al. 2020). Nevertheless, differences in spatial scale and study design can complicate direct comparisons between sampling methods, particularly when assessing the relative performance of trapping and owl pellet analysis. In addition, a major limitation of such large‐scale syntheses is that they require the combined analysis of datasets collected in very different habitats and associated with several owl species with distinct hunting strategies (Luiselli and Capizzi 1996; McDonald et al. 2014; Schoenefuss et al. 2024). This heterogeneity makes it difficult to identify clear relationships between owl diet and small mammal community composition and complicates direct comparisons between the two sampling methods. Regional‐scale comparisons that minimise climatic and biogeographic variability, while maintaining sufficient ecological heterogeneity, remain particularly scarce.

Small mammal communities are widely used as indicators of environmental conditions. Consequently, their remains are also important proxies in paleoenvironmental reconstructions that help to understand past ecosystems and climate change. Owl pellets are an important source of small mammal remains in many fossil cave assemblages, particularly in Late Pleistocene deposits (Andrews 1990). Several studies have shown that the species composition of owl pellet assemblages reflects the habitat preferences and ecological needs of small mammals (Dodson and Wexlar 1979; Lopez 2020). Small mammals are valuable environmental indicators because many species react sensitively to climatic and habitat changes (Rowe and Terry 2014). Fossilised small mammals from owl pellets have been extensively used to infer past environmental conditions and reconstruct regional paleoenvironmental contexts (Ortiz and Jayat 2012; Royer et al. 2020).

Since biodiversity and paleoenvironmental studies rely on small mammal abundance in pellets, identifying potential biases from owl prey selection is crucial to ensure accurate representation of the natural mammal community. Despite the widespread use of trapping and pellet analysis, direct comparisons based on synchronously collected datasets appear to be relatively rare (Heisler et al. 2016; Schoenefuss et al. 2024; Torre et al. 2004), which limits our ability to evaluate how methodological differences influence the observed structure of small mammal communities. This study presents a regional comparison of conventional small mammal trapping and owl pellet analysis using datasets collected simultaneously at the same locations across Central Europe. Our aim was to evaluate the relative effectiveness of the two sampling approaches for detecting small mammal diversity and to examine how predator selectivity may influence the representation of small mammal communities in pellet assemblages. We expected that pellet analysis could detect higher taxonomic diversity, potentially reflecting the broader spatial integration of prey by owls, but species abundances inferred from pellets may differ from those recorded by trapping. At the same time, it should be noted that while species such as the western barn owl have cosmopolitan distributions spanning highly diverse global biomes, this study is explicitly restricted to Central European temperate ecosystems. Our primary objective is not to derive generalisations about the foraging behaviour or prey selection of these birds across their entire geographic range. Rather, this study evaluates the regional effectiveness, benefits and limitations of owl pellet analysis compared to conventional live‐trapping, with a focus on monitoring small mammal communities and reconstructing local species assemblages within this biogeographically coherent region.

## 2 Materials and Methods

### 2.1 Data Collection

In this study, we examined small mammal communities from Central Europe using two methods: live trapping and owl pellet analysis. The region, characterised by relatively uniform climate and vegetation (Chytrý et al. 2022; Hoffman 1983), has a long‐standing tradition of pellet‐based faunal studies (Kretzoi 1964; Schmidt and Sipos 1971), facilitating comparative research. Data were compiled from 26 published studies and supplementary unpublished field reports, resulting in a comprehensive dataset of 40 case studies across eight countries (Hungary, Serbia, Ukraine, Romania, Slovakia, the Czech Republic, Poland and Croatia) (Figure 1). Individual ‘cases’ were defined as independent sampling units derived from published studies. In studies reporting data across multiple years or sampling periods, each year was treated as a separate case when sample size was sufficient for statistical analyses. Only cases with adequate sample sizes were included to ensure robust and comparable results. In each case, trapping and pellet collection were conducted at the same or nearby locations and within comparable sampling periods, providing a consistent basis for cross‐site comparison. Such spatial and temporal alignment is not always achieved in regional‐scale ecological studies. The compiled dataset therefore offers a relatively large and systematically processed set of comparable case studies within a geographically coherent region. In multi‐locality datasets, analyses were restricted to sampling sites representing comparable habitat types. The main characteristics of the reviewed case studies are summarised in Table 1. Live trapping utilised Sherman live traps or pitfall traps across seven habitat types: coniferous and deciduous forests, bogs/swamps, wet meadows, dry grasslands, mosaic landscapes and agricultural areas.

**FIGURE 1:**
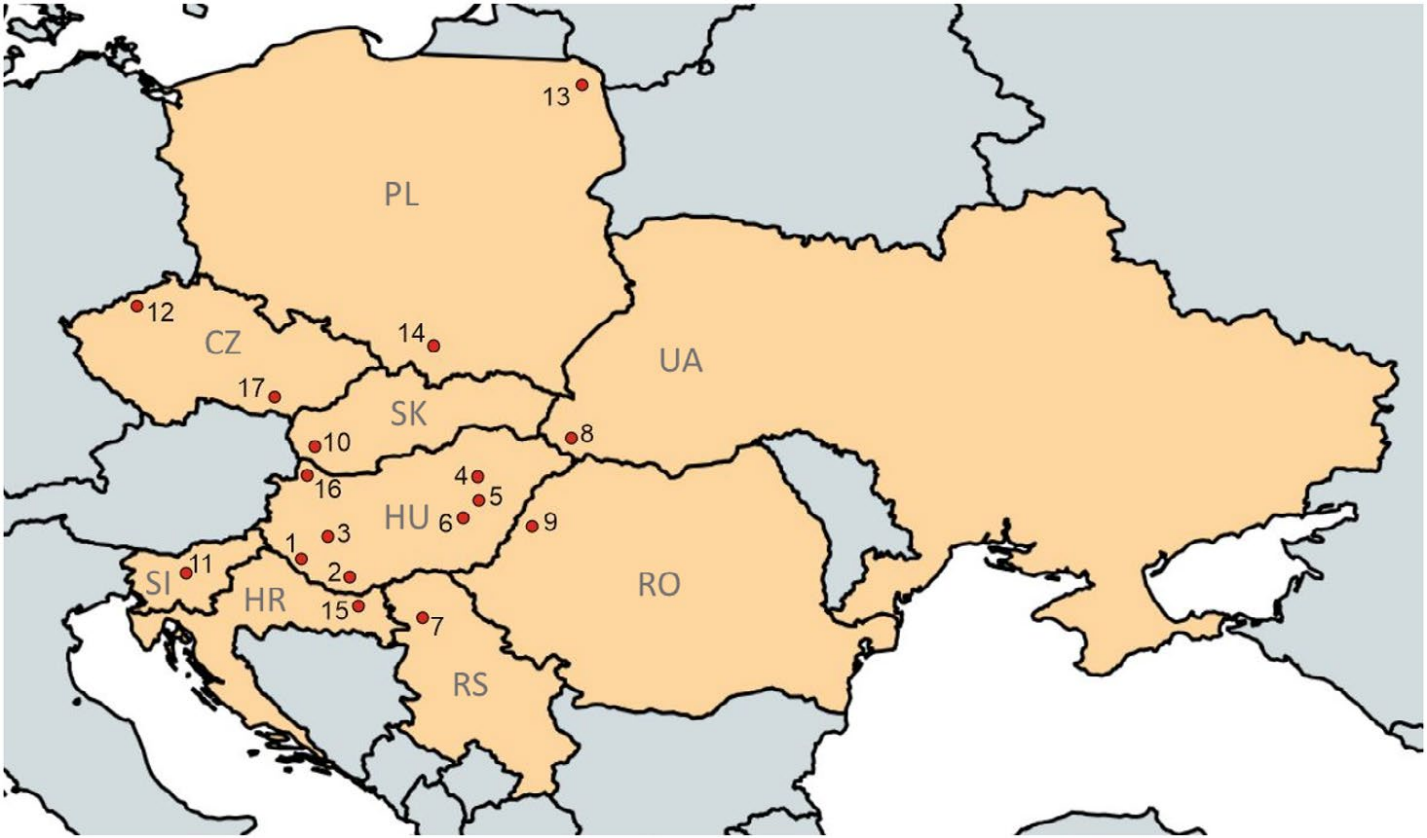
Geographic distribution of the analysed case studies. Each point represents a study location. For multi‐locality datasets, points indicate the approximate centre of the study area. The sites are numbered in accordance with the reference site numbers shown in Table 1 for the case studies.

**TABLE 1:** Overview of the datasets included in the analysis.

Case	Ref. Pellet	Ref. Trap	Country	Ref. no. in Figure 1	Owl species	Study habitat
1	Purger (2016)	Horváth et al. (2004)	Hungary	1	Barn owl *(Tyto alba)*	Deciduous forest
2	Purger (2016)	Horváth et al. (2004)	Hungary	1	Barn owl *(Tyto alba)*	Deciduous forest
3	Horváth (1998a)	Horváth (1998b)	Hungary	2	Barn owl *(Tyto alba)*	Meadow
4	Purger (2014)	Lanszki (2004)	Hungary	3	Barn owl *(Tyto alba)*	Moor/Marshland
5	Purger (2014)	Lanszki et al. (2008)	Hungary	3	Barn owl *(Tyto alba)*	Moor/Marshland
6	Cserkész and Gubányi (2008)	Cserkész and Gubányi (2008)	Hungary	4	Barn owl *(Tyto alba)*	Dry grassland
7	Cserkész and Gubányi (2008)	Cserkész and Gubányi (2008)	Hungary	4	Barn owl *(Tyto alba)*	Dry grassland
8	Cserkész and Gubányi (2008)	Cserkész and Gubányi (2008)	Hungary	4	Barn owl *(Tyto alba)*	Dry grassland
9	Unpublished data	Unpublished data	Hungary	4	Barn owl *(Tyto alba)*	Dry grassland
10	Unpublished data	Unpublished data	Hungary	4	Barn owl *(Tyto alba)*	Dry grassland
11	Unpublished data	Unpublished data	Hungary	4	Barn owl *(Tyto alba)*	Dry grassland
12	Unpublished data	Unpublished data	Hungary	4	Barn owl *(Tyto alba)*	Dry grassland
13	Unpublished data	Unpublished data	Hungary	4	Barn owl *(Tyto alba)*	Dry grassland
14	Unpublished data	Unpublished data	Hungary	4	Barn owl *(Tyto alba)*	Dry grassland
15	Unpublished data	Unpublished data	Hungary	4	Barn owl *(Tyto alba)*	Dry grassland
16	Unpublished data	Unpublished data	Hungary	4	Barn owl *(Tyto alba)*	Dry grassland
17	Unpublished data	Unpublished data	Hungary	4	Barn owl *(Tyto alba)*	Dry grassland
18	Unpublished data	Unpublished data	Hungary	4	Barn owl *(Tyto alba)*	Dry grassland
19	Unpublished data	Unpublished data	Hungary	4	Barn owl *(Tyto alba)*	Dry grassland
20	Unpublished data	Unpublished data	Hungary	4	Barn owl *(Tyto alba)*	Dry grassland
21	Unpublished data	Unpublished data	Hungary	4	Barn owl *(Tyto alba)*	Dry grassland
22	Endes and Dudás (2017)	Tóth (2014)	Hungary	5	Barn owl *(Tyto alba)*	Dry grassland
23	Endes and Dudás (2017)	Mérő and Bocz (2012)	Hungary	5	Barn owl *(Tyto alba)*	Dry grassland
24	Unpublished data	Unpublished data	Hungary	6	Barn owl *(Tyto alba)*	Meadow
25	Bjelić‐Čabrilo (2006)	Bjelić‐Čabrilo (2006)	Serbia	7	Barn owl *(Tyto alba)*	Agricultural land
26	Bjelić‐Čabrilo (2006)	Bjelić‐Čabrilo (2006)	Serbia	7	Long‐eared owl *(Asio otus)*	Agricultural land
27	Zahorodnyi et al. (2021)	Barkaszi et al. (2021)	Ukraine	8	Little owl *(Athene noctua)*	Mosaic landscape
28	Milca et al. (2013)	Benedek and Sirbu (2009)	Romania	9	Barn owl *(Tyto alba)*	Mosaic landscape
29	Milca et al. (2013)	Benedek and Sirbu (2009)	Romania	9	Long‐eared owl *(Asio otus)*	Mosaic landscape
30	Baláz and Ambros (2005)	Baláz and Ambros (2005)	Slovakia	10	Barn owl *(Tyto alba)*	Deciduous forest
31	Baláz and Ambros (2005)	Baláz and Ambros (2005)	Slovakia	10	Long‐eared owl *(Asio otus)*	Deciduous forest
32	Tome (1991)	Tome (1991)	Slovenia	11	Long‐eared owl *(Asio otus)*	Moor/Marshland
33	Tome (1991)	Tome (1991)	Slovenia	11	Long‐eared owl *(Asio otus)*	Moor/Marshland
34	Tome (1991)	Tome (1991)	Slovenia	11	Long‐eared owl *(Asio otus)*	Moor/Marshland
35	Zárybnická et al. (2013)	Zárybnická et al. (2013)	Czech Republic	12	Boreal owl *(Aegolius funereus)*	Evergreen forest
36	Zawadzka and Zawadzka (2007)	Zárybnická et al. (2013)	Poland	13	Tawny owl *(Strix aluco)*	Evergreen forest
37	Bochenski (1990)	Bochenski (1990)	Poland	14	Tawny owl *(Strix aluco)*	Deciduous forest
38	Szép (2020)	Horváth et al. (2008)	Croatia	15	Barn owl *(Tyto alba)*	Deciduous forest
39	Jánoska (1995)	Németh (2014)	Hungary	16	Barn owl *(Tyto alba)*	Deciduous forest
40	Gaisler et al. (1996)	Gaisler et al. (1996)	Czech Republic	17	Long‐eared owl *(Asio otus)*	Deciduous forest
41	Gaisler et al. (1996)	Gaisler et al. (1996)	Czech Republic	17	Tawny owl *(Strix aluco)*	Deciduous forest
42	Gaisler et al. (1996)	Gaisler et al. (1996)	Czech Republic	17	Eurasian eagle‐owl *(Bubo bubo)*	Deciduous forest

*Note:* Each case (Case 1–Case 42) corresponds to a specific published study and represents a paired dataset. In these case studies small mammal communities were assessed using both live trapping and owl pellet analysis at the same location and during the same sampling period. The table includes the original references for pellet and trapping data, country, owl species and study habitat. Each case (Case 1–Case 42) corresponds to a specific published study and represents a paired dataset in which small mammal communities were assessed using both live trapping and owl pellet analysis at the same location and during the same sampling period. The table includes the original references for pellet and trapping data, country, owl species and study habitat. The locations of the case study sites are shown in Figure 1 and are labelled according to the reference numbers listed in the table.

Pellet analyses included specimens from six owl species: western barn owl ( *Tyto alba* ), long‐eared owl ( *Asio otus* ), tawny owl ( *Strix aluco* ), little owl ( *Athene noctua* ), boreal owl ( *Aegolius funereus* ) and Eurasian eagle‐owl ( *Bubo bubo* ). The barn owl, which is the most frequently studied species, was emphasised due to its prevalence in open habitats and its relatively generalist diet in such environments (Salvati et al. 2002; Shawyer 1994; Taylor 2003). This dataset enabled statistical comparison of species composition between the two sampling techniques, providing insight into their respective biases and complementarities in monitoring small mammal assemblages.

To avoid bias in comparing Sherman trap data with pellet samples, non‐trappable species (e.g., Chiroptera, *Mustela erminea* , *Mustela nivalis* , *Sciurus vulgaris* , *Erinaceus concolor* ) and records without species‐level identification were excluded. *Apodemus flavicollis* , *Apodemus sylvaticus* and *Apodemus uralensis* were pooled due to identification challenges, as were *Mus musculus* and *Mus spicilegus* .

In Hungary, conservation of the endangered Hungarian birch mouse (*Sicista trizona trizona*) in the Borsodi‐Mezőség area has involved pitfall trap monitoring since 2006 (Cserkész 2020). From 2006 to 2009, Sherman traps, pitfall traps and *Tyto alba* pellet analyses were used simultaneously (Cserkész 2007). Since 2010, pitfall trapping and owl pellet analysis have been combined in monitoring programs, enabling direct comparison of small mammal assemblages between methods (Cserkész 2017).

### 2.2 Statistical Analyses

Sampling efficiency was assessed using three diversity indices alongside multivariate analyses to compare methods. To control for sample size effects, species richness was standardised via Shareholder Quorum Subsampling (SQS) at a quorum of 0.8. Unlike rarefaction, which can underestimate diversity in highly diverse samples, SQS estimates diversity based on sample coverage, offering a more accurate measure of community diversity (Alroy 2010; Viteri et al. 2022). Fisher's exact test (*t*) was applied for data with a standard normal distribution, while the Mann–Whitney test (*w*) was used for non‐normally distributed data to assess significant differences between conventional trapping and owl pellet analysis.

Taxon evenness was calculated by using the Probability of Interspecific Encounter (PIE), a metric ranging from 0 to 1. Higher PIE values indicate higher evenness. The advantage of PIE is its independence from sample size, assuming a sufficient number of individuals per sample (Hurlbert 1971). Species dominance was assessed by using Simpson's diversity index (1‐D), indicating the probability that two randomly chosen individuals from the same sample belong to the same species; higher values correspond to lower dominance (Somerfield et al. 2008).

Community composition differences between sampling methods were analysed using the Bray–Curtis dissimilarity matrix, calculated from species abundance data. Differences among methods were statistically tested by using the non‐parametric Analysis of Similarity (ANOSIM, Clarke and Green 1988). Additionally, the Mann–Whitney test was applied to verify significant differences in species composition among groups.

Non‐metric multidimensional scaling (NMDS) ordination, based on rank‐transformed Bray‐Curtis dissimilarities, was used for the graphical representation of community differences. NMDS is a robust ordination technique suitable for ecological data, as it does not assume linear relationships and effectively captures non‐linear patterns (Clarke 1993; Clarke and Warwick 2001). The accuracy of the NMDS ordination was assessed by using a stress function, where lower stress values indicate that the graphical representation accurately reflects the original dissimilarities between samples.

Finally, to identify species strongly associated with specific sampling methods, the Indicator Value Index (IndVal) was calculated, highlighting the characteristic small mammal taxa indicative of each sampling approach.

All analyses and visualisations were performed using R statistical software (version 4.4.1; R Core Team 2024), with the packages divDyn (Kocsis et al. 2019), vegan (Oksanen et al. 2022) and indicspecies (De Cáceres and Legendre 2009).

## 3 Results

Each dataset analysed in this study is referred to as a ‘case’ (Table 1). Each case corresponds to a paired dataset from a specific study, in which live trapping and owl pellet collection were conducted at the same location and during the same sampling period. Cases are numbered sequentially (Case 1–Case 42) and correspond directly to the studies listed in Table 1.

Owl pellet samples showed higher diversity compared to all methods used to measure sampling efficiency (Table 2). The high values of Richness (*x̄*_pellet_ = 11.6 ± 0.533 SE, *x̄*_trap_ = 6.69 ± 0.444 SE, *t* = 7.14, *p* < 0.001), Simpson's diversity index (*x̄*_pellet_ = 0.646 ± 0.0253 SE, *x̄*_tra*p*_ = 0.550 ± 0.0343 SE, *w* = 806, *p* = 0.02) and PIE (*x̄*_pellet_ = 0.665 ± 0.0260 SE, *x̄*_tra*p*_ = 0.550 ± 0.0343 SE, *w* = 860, *p* = 0.003) indicate that owl pellet analysis captures a broader spectrum of small mammal species within the analysed datasets than trapping methods do (Figure 2). The results were significant in all comparisons.

**TABLE 2:** Summary of diversity metrics (species richness, PIE—Probability of Interspecific Encounter, and Simpson's (1‐D) index) for Sherman trap (T) and pellet (P) datasets.

	Richness P	Richness T	PIE P	PIE T	Simpson P (1‐D)	Simpson T (1‐D)
Case 01	16	7	0.749773845	0.318673045	0.749773845	0.318673045
Case 02	16	8	0.788412152	0.352761513	0.788412152	0.352761513
Case 03	14	11	0.878521245	0.745356125	0.878521245	0.745356125
Case 04	17	9	0.842823594	0.771332587	0.842823594	0.771332587
Case 05	17	9	0.861245509	0.485642946	0.861245509	0.485642946
Case 06	14	7	0.809942663	0.631316159	0.809942663	0.631316159
Case 07	12	9	0.666530616	0.686117038	0.666530616	0.686117038
Case 08	13	7	0.587834512	0.544946084	0.587834512	0.544946084
Case 22	12	5	0.78577744	0.592002962	0.78577744	0.592002962
Case 23	8	10	0.358148264	0.659778092	0.358148264	0.659778092
Case 24	14	2	0.697662286	0.458333333	0.697662286	0.458333333
Case 25	13	5	0.846448272	0.526705447	0.846448272	0.526705447
Case 26	9	5	0.655048178	0.526705447	0.655048178	0.526705447
Case 27	9	7	0.589808154	0.720868347	0.589808154	0.720868347
Case 28	7	5	0.776915356	0.509009009	0.776915356	0.509009009
Case 29	5	5	0.426148542	0.509009009	0.426148542	0.509009009
Case 30	5	4	0.270390071	0.67006456	0.270390071	0.67006456
Case 31	13	4	0.734366036	0.67006456	0.734366036	0.67006456
Case 32	6	6	0.507758001	0.633906634	0.507758001	0.633906634
Case 33	6	3	0.565796614	0.113570181	0.565796614	0.113570181
Case 34	6	4	0.280932112	0.116405599	0.280932112	0.116405599
Case 35	14	8	0.730672723	0.68019192	0.730672723	0.68019192
Case 36	12	7	0.840199643	0.794399469	0.840199643	0.794399469
Case 37	10	5	0.841632653	0.143350009	0.841632653	0.143350009
Case 38	18	5	0.584446	0.520833109	0.584446	0.520833109
Case 39	8	7	0.440124249	0.68443781	0.440124249	0.68443781
Case 40	8	10	0.231468736	0.625489225	0.231468736	0.625489225
Case 41	9	10	0.666427225	0.625489225	0.666427225	0.625489225
Case 42	11	10	0.688975642	0.625489225	0.688975642	0.625489225

*Note:* Richness, species richness, the number of species found in a community; PIE, Probability of Interspecific Encounter: The probability that two randomly captured individuals belong to different species (Hurlbert index), Simpson (1‐D), Simpson Diversity Index: The probability that two randomly selected individuals belong to different species, *P*, owl pellet, T, Sherman trap.

**FIGURE 2:**
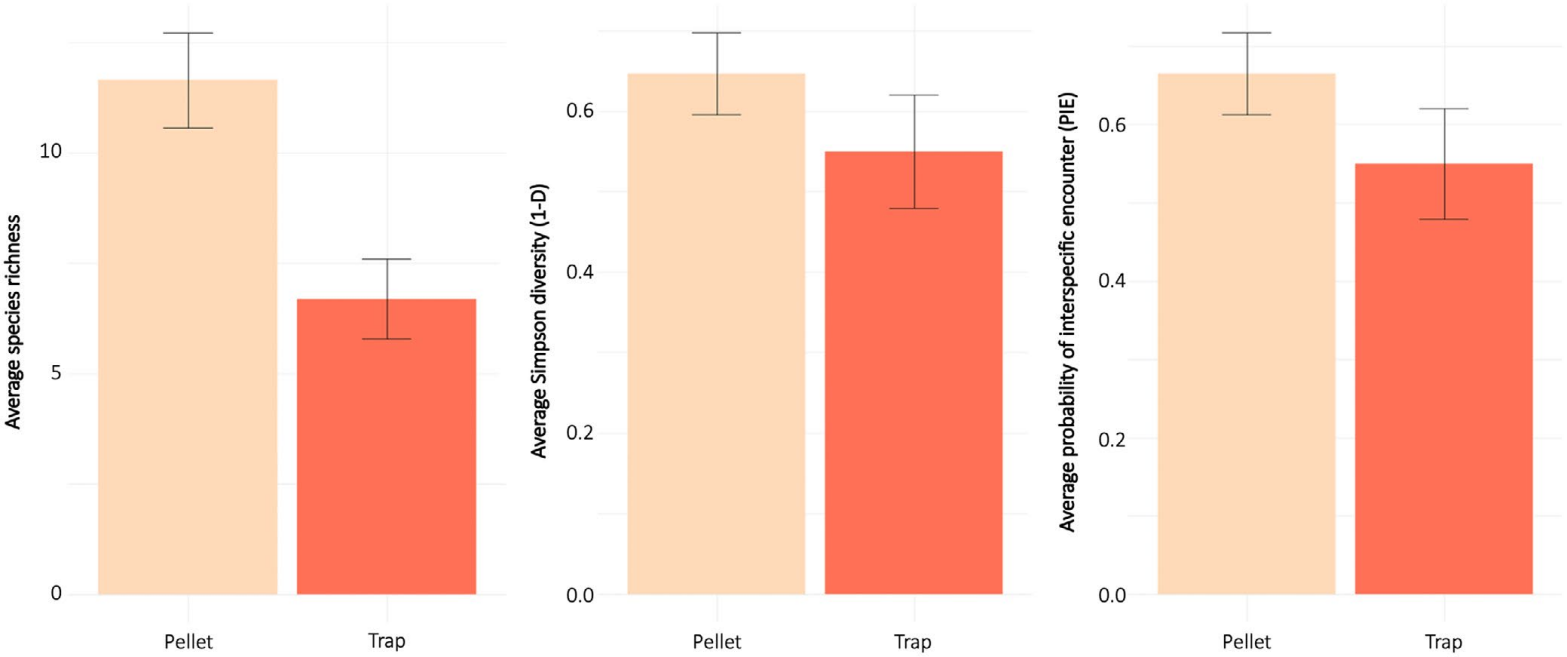
Diversity indices of Sherman trap and pellet datasets. Mean species richness, Simpson's diversity (1‐D) ± SE and probability of interspecific encounter (PIE) ± SE calculated from owl pellet and small mammal trap samples.

Diversity calculated from the SQS analysis indicates a high level of sample coverage (almost 100%) within the pellet dataset. The estimated species richness is therefore relatively stable. Fewer species were detected by the trapping method, and the coverage was lower (95% average), indicating that not all the species were detected by this approach (Figure 3). The standard deviation of the confidence intervals was higher for trapping than for pellet analysis, indicating greater uncertainty in the SQS estimations.

**FIGURE 3:**
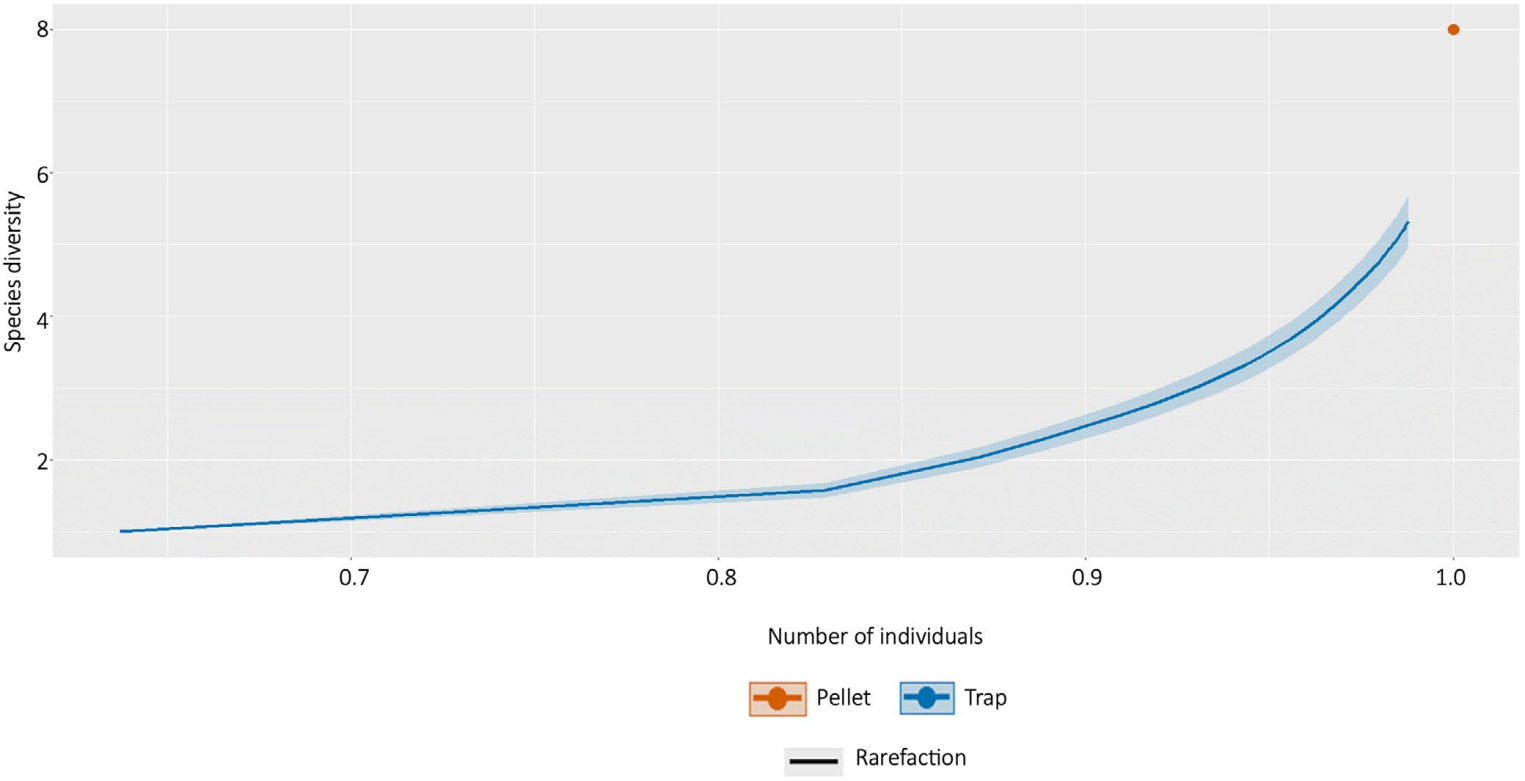
The diversity curve was calculated by using SQS at 95% coverage. The blue band represents the 95% confidence interval.

The composition of the small mammal communities inferred from pellet analysis and trapping differed, with some overlap based on similarity analysis (ANOSIM *R* = 0.293), indicating significant differences between methods (*p* < 0.0001). The stress value indicates a good ordination with minimal risk of misinterpretation (Stress = 0.115, non‐metric fit *r* ^2^ = 0.986, Linear fit *R* ^2^ = 0.914).

The NMDS ordination illustrates that the two sampling methods show differences in small mammal community composition. While the pellet and trap data from open areas are concentrated in overlapping parts of the point clouds, the small mammal composition of the forest traps differs from the community composition inferred from the owl pellet collected at the same time (Figure 4).

**FIGURE 4:**
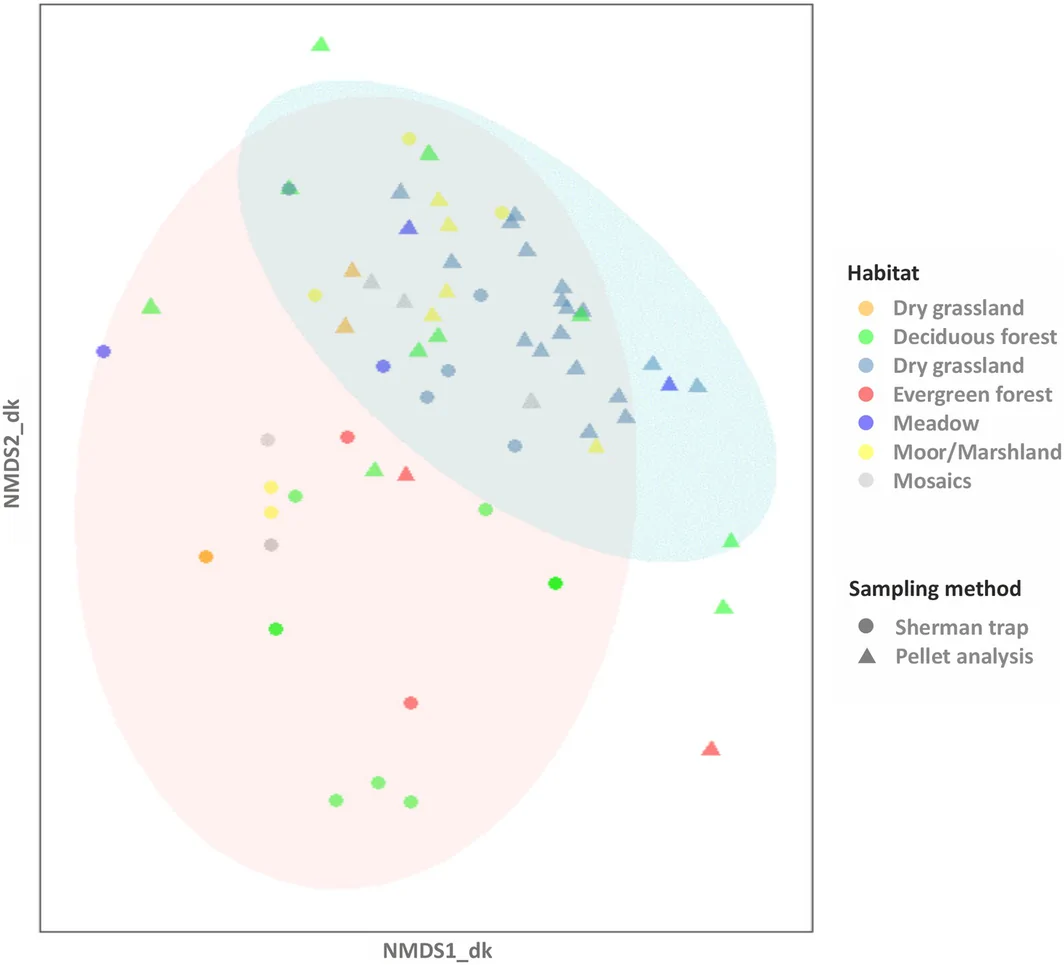
NMDS ordination of small mammal community composition based on Bray–Curtis dissimilarity. Differences between sampling methods are indicated by symbol type, while habitat types are represented by colours.

The IndVal test results showed that the bank vole ( *Clethrionomys glareolus* ) was the dominant species in the Sherman traps placed in forests. The community structure based on trap data was significantly influenced by the dominance of *Clethrionomys glareolus* (*p* = 0.0014). However, more species seemed to have an influential effect on the community based on the owl pellet analysis. These include the harvest mouse, *Micromys minutus* (*p* = 0.0006); members of the genus *Mus* (*p* = 0.0007), the common shrew, *Sorex araneus* (*p* = 0.0015); the bicoloured shrew, *Crocidura leucodon* (*p* = 0.0011); the lesser white‐toothed shrew, *Crocidura suaveolens* (*p* = 0.0012); the European water vole, *Arvicola amphibius* (*p* = 0.0022); and the common vole, *Microtus arvalis* (*p* = 0.0013). In the IndVal test results, small mammal species typical of forest habitats were underrepresented in the pellets compared to the trapping data.

The species composition of the Arvicolinae and Soricinae populations sampled by pitfall traps was significantly different from that of the *Tyto alba* pellets (*R* = 0.51, *p* = 0.0001). Evaluation of the stress function provided a clear representation without ambiguity (Stress = 0.05, Non‐metric fit *R* ^2^ = 0.997, Linear fit *R* ^2^ = 0.988). There are statistically significant differences in the relative abundance of the different species. Faunal composition estimated from pitfall traps and owl pellet analysis differs. *Microtus arvalis* was found to be more abundant in pellet samples than in trapping data, indicating the common vole was the main prey of *Tyto alba* at the study site. The proportion of *Crocidura suaveolens* and *C. leucodon* in the diet of *Tyto alba* was also higher than expected. However, *Sorex araneus* and *S. minutus* were underrepresented in the barn owl pellets (Figure 5B). At the same time, pitfall trapping proved to be the most effective method of monitoring *Sicista trizona*, which exhibited lower abundance in owl pellets compared to trapping.

**FIGURE 5:**
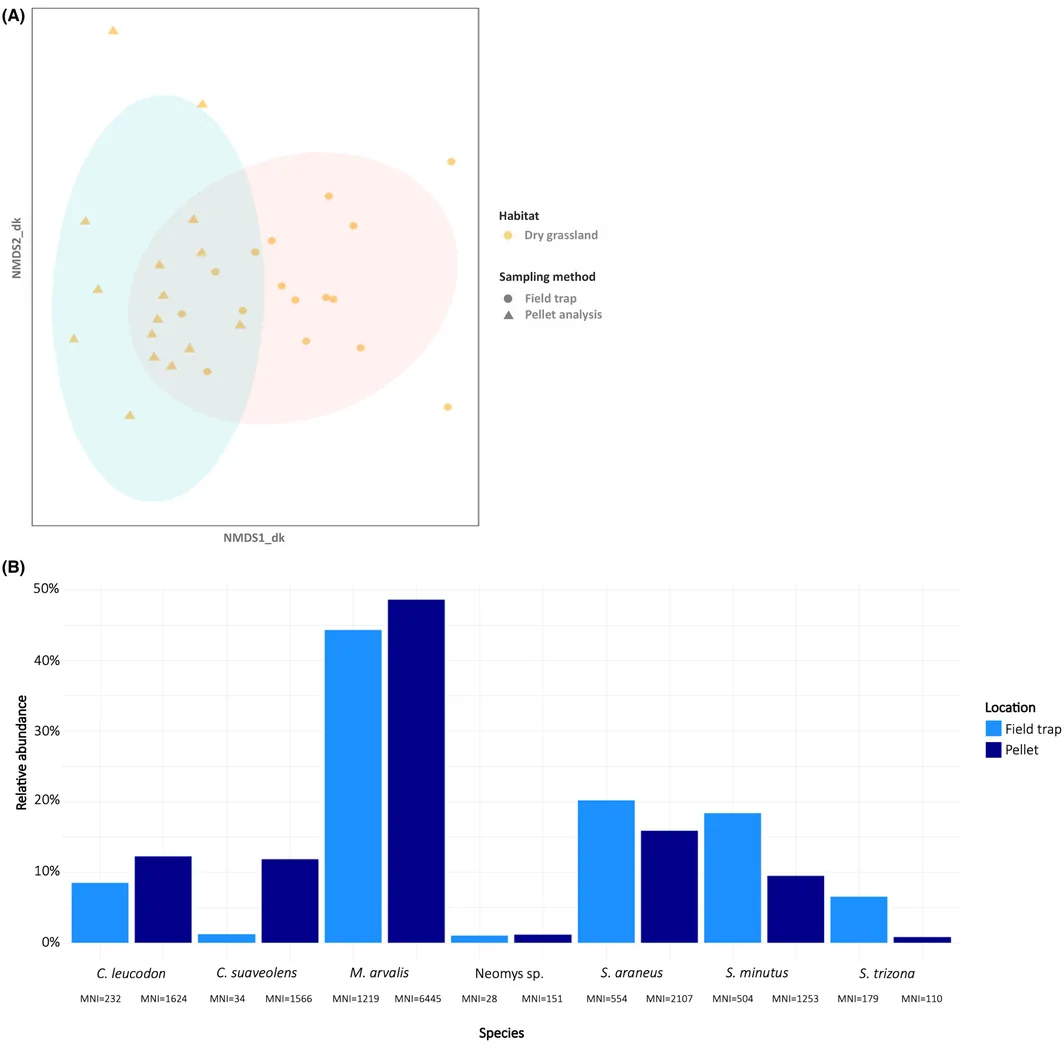
Comparison of barn owl ( *Tyto alba* ) pellet analysis and conventional trapping in the Borsodi‐Mezőség (Hungary) between 2006 and 2022. (A) NMDS ordination of small mammal community composition based on Bray–Curtis dissimilarity, showing differences between pellet‐derived and trapping datasets. (B) Relative abundance (%) of small mammal species in pellet samples (dark blue) and trapping data (light blue).

## 4 Discussion

### 4.1 Comparison of Owl Pellet Analysis and Conventional Trapping

Owl pellet analysis can reveal a wider range of local small mammal diversity than conventional trapping methods, particularly when sampling coverage is limited. Similar patterns have been reported from a wide range of regions and ecosystems, where owl pellet assemblages often detected higher small mammal diversity than conventional trapping methods (Debus et al. 2004; Laurie 1971; Rocha et al. 2011). Pellet samples also contained a higher number of species, including taxa that are often rarely recorded in trapping surveys, which is consistent with previous studies suggesting that pellet analysis can complement conventional trapping approaches in the study of small mammal communities (Avenant 2005; Balčiauskienė 2005; Heisler et al. 2016; Luiselli and Capizzi 1996). Similar patterns have been reported from many regions outside Europe, where owl pellet assemblages have been shown to be effective for documenting small mammal diversity and, in some cases, detecting species that are rarely captured in trapping surveys (Bonvicino and Bezerra 2003; Magrini and Facure 2008; Rocha et al. 2011). Therefore, these results suggest that pellet analysis can be an effective complementary approach for assessing small mammal diversity across broader spatial and temporal scales than conventional trapping.

The results of ANOSIM analysis indicate significant differences in small mammal community composition between the two sampling methods, suggesting that pellet‐derived data and trapping surveys may represent partially different components of the local fauna. Trap captures are influenced by trap type, bait and species‐specific behaviour, which can affect the representation of species in trapping surveys (Stephens and Anderson 2014; Torre et al. 2004). Additionally, trapping surveys are typically conducted over relatively short sampling periods and within defined sampling areas, which may limit their spatial and temporal coverage. In contrast, owls forage over larger areas and may integrate prey remains from a broader landscape around the roost (Taylor 1994; Grzywaczewski 2009; Kasprzykowski and Golawski 2006; Martínez et al. 2003). Therefore, pellet assemblages may help to reduce some of the spatial and temporal limitations associated with trapping surveys. The SQS analysis showed complete sample coverage in the pellet dataset, whereas trapping surveys displayed lower coverage (Figure 3). Rarefaction curves further suggest that pellet assemblages approach asymptotic diversity with fewer samples than trapping surveys (Figure 6).

**FIGURE 6:**
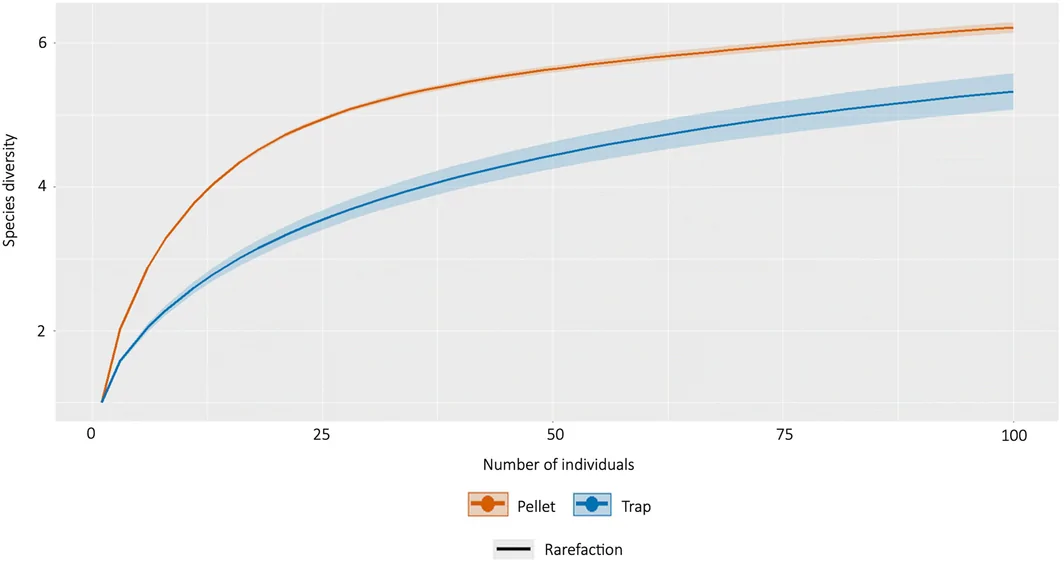
Rarefaction curves showing species richness as a function of sample size. Pellet samples (orange) reach higher richness at lower sampling effort than trapping data (blue). Shaded areas represent 95% confidence intervals.

The IndVal analysis indicates further differences in the small mammal assemblages recorded by pellet analysis and trapping surveys. In the trapping dataset, the forest‐dwelling *Clethrionomys glareolus* was the dominant species, whereas it was much less frequently recorded in the pellet assemblages. Consistent with previous studies (Horváth and Horváth 2024; Korpimäki et al. 2002), the high abundance of *Microtus arvalis* , which dominated the pellet assemblages, is likely reflected in open habitats commonly used by barn owls for hunting. These patterns suggest that owl pellet analysis can provide useful information on habitat‐related variation in small mammal communities across broader landscapes. However, owls forage across a range of habitats (Marti 1974; Séchaud et al. 2021), so they may sample prey from a broader spatial area than trapping surveys, which are usually carried out in specific habitat patches. Many studies report that barn owls frequently hunt in open habitats (Getz 1961; Southern and Lowe 1968; Torre et al. 2004), which may lead to the underrepresentation of forest‐associated species in pellet assemblages (Van Strien et al. 2015; Yom‐Tov and Wool 1997). Some other owl species, like *Asio otus* and *Bubo bubo* , use open patches within forested landscapes and ecological corridors as hunting grounds (Wijnandts 1984; Mérő et al. 2021). Similarly, a study from Hungary reported that the diets of *Tyto alba* and *Strix aluco* contained a high proportion of species associated with open habitats (Kalivoda 1994). Therefore, pellet analysis alone may provide an incomplete representation of local small mammal communities. Prey selectivity has been documented in several owl species; for example, *Asio otus* has been reported to avoid shrews, possibly due to their taste or odour (Birrer 2009; Yom‐Tov and Wool 1997). Such selective predation may influence pellet‐based monitoring results and contribute to differences between pellet‐derived assemblages and trapping data (Stephens and Anderson 2014; Torre et al. 2004).

### 4.2 Bias in Barn Owl Pellets Based on the Sicista Case Study

A similar pattern was reported during a conservation monitoring program for the endangered *Sicista trizona*, where data collection has been ongoing since 2006 at its only known Hungarian locality. Two primary methods—pitfall traps and analysis of barn owl pellets—have been employed (Cserkész 2017). While both methods yielded an identical species composition, differences emerged in relative abundances. Pitfall traps are often effective at capturing a broad range of small mammal species, including rare taxa and juveniles, although capture success may vary among species and trap designs (Umetsu et al. 2006). For example, acrobatic, agile or more wary species, like *Apodemus* wood mice, may avoid these traps (Cserkész and Gubányi 2008). It was expected that the diet of the barn owl would broadly reflect the composition of the local small mammal community present in this open habitat. However, ANOSIM results using the Bray‐Curtis index revealed significant differences between the two datasets. Barn owl pellets contained higher proportions of species characteristic of dry, open areas, such as *Microtus arvalis* , *Crocidura leucodon* and *C. suaveolens* . In contrast, red‐toothed shrews, favouring moister habitats, were underrepresented in pellets. This pattern may reflect the tendency of barn owls to forage more frequently in the driest and most open areas of their habitat. It also suggests that the feeding preferences of *T. alba* are not solely based on the availability of their prey species (Cserkész 2017).

In our study area, *Microtus arvalis dominated in barn owl pellets*, suggesting that this species formed a major component of the barn owl diet at that site. Barn owls may also behave as opportunistic predators. Under unfavourable conditions, they tend to consume a wider range of small mammal species that provide lower energetic returns (Bernard et al. 2010; Romano et al. 2020). In addition to small mammals, barn owl pellets occasionally contained remains of other prey groups, including birds, reptiles, amphibians and insects. Consequently, pellet assemblages may not fully represent the relative composition of local small mammal communities (Figure 5A).

### 4.3 Evaluation of Bias by the Owls

Although owls may exhibit prey preferences, many taxa in the local small mammal community are typically represented in their pellet remains (Mikkola 1983). However, the relative abundance of species in pellets does not always reflect true population structure (Comay and Dayan 2018b). Studies indicate a stronger correlation between pellet composition and actual community structure in open habitats (Terry 2009, 2010). In contrast, heterogeneous landscapes with both open and closed habitats introduce variability due to habitat‐specific prey availability and owl foraging behaviour (Sadykova and Smirnov 2005).

Many owl species may exhibit prey selectivity; nevertheless, opportunistic behaviour can result in increased consumption of alternative prey when access to preferred species is restricted (Balčiauskienė and Naruševičius 2006; Tores et al. 2005). Species that are smaller, larger or inhabit less accessible habitats may be underrepresented in owl diets (Korpimäki 1992). While these patterns are well established, several studies have demonstrated that owl pellet assemblages may deviate from actual small mammal community composition due to predator selectivity and habitat‐dependent foraging behaviour (Andrews 1990; Terry 2010). For instance, long‐term analyses of *Tyto alba* diets in Central European grassland habitats revealed notable discrepancies between pellet‐derived and observed community structures (Torre et al. 2015). Similar mismatches have been reported from other regions, indicating that even in relatively homogeneous environments, diet‐based monitoring may introduce bias. These findings highlight the need for careful evaluation of pellet data when used as a proxy for biodiversity assessment (Bellocq 1998; Brown et al. 1988; Dickman et al. 1991). However, it is also necessary to underline that focusing on owl prey limitations (bias in focus toward certain prey types) per se does not adequately acknowledge the limitations of other techniques (e.g., different types of trapping surveys) and how they may also misrepresent the real relative abundances of the given habitat and site. Thus, the best way forward in such cases is to perform multiple sampling or monitoring techniques wherever possible. Furthermore, the predator–prey dynamics and methodological biases identified in this synthesis must be interpreted strictly within the ecological context of Central Europe. Cosmopolitan predators, particularly *Tyto alba* , display substantial behavioural plasticity and context‐dependent prey preferences across contrasting global biomes–ranging from Australian arid zones and open eucalypt forests to Neotropical savannas. Consequently, the foraging tactics, habitat use and prey selectivity observed in Central European agricultural and forest mosaics cannot be directly extrapolated to other continents or ecozones where prey community structures, vegetation density and seasonal prey availability differ fundamentally. Caution is therefore warranted when broadening behavioural inferences, as owl‐derived sampling biases are strongly shaped by regional ecological conditions.

### 4.4 Implications for Paleoenvironmental Reconstructions

The results of the present investigations also have implications for paleoenvironmental reconstructions. The potential selectivity of owls among available small mammal prey represents an important consideration not only for biodiversity monitoring but also for paleoenvironmental reconstruction. The Plio‐Pleistocene cave sediments used to reconstruct paleoenvironments have, in many cases, been shown to be the result of the accumulation of owl pellets (Andrews 1995; Comay and Dayan 2018b; Pinero et al. 2016). Studies of owl hunting behaviour have shown discrepancies between the small mammal assemblages found in owl pellets and the structure of living small mammal communities, indicating the need for caution when interpreting fossil assemblages derived from owl pellet accumulations. The hunting range and prey preferences of owls may have also influenced the composition of small mammal assemblages in the Pliocene and Pleistocene. Because paleoenvironmental reconstructions often rely on the relative abundance of small mammal taxa, predator‐related biases should be carefully considered when interpreting fossil assemblages. At the same time, such assemblages may still preserve meaningful ecological signals when evaluated within an appropriate taphonomic framework.

## 5 Conclusion

The comprehensive analysis of the diet of owls in the Central European region has allowed us to demonstrate that owl pellet analysis is an effective method for determining the species composition of a small mammal community in terms of diversity indicators. However, owl prey preferences may influence the species composition derived from pellets, and the relative abundance of species recorded in pellets may differ from that obtained by trapping surveys. Nevertheless, further studies of hunting ranges and food preferences of owls are needed to understand the extent and nature of this bias in the cases of different owl species. A better understanding of the problem is important for more effective biodiversity monitoring, a priority in the current biodiversity crisis. Given that wide‐ranging owl species adapt their hunting strategies to local environments globally, future cross‐regional studies are also needed to determine whether the methodological limitations and detection probabilities identified here in Central Europe hold in ecosystems on other continents with distinct prey dynamics. A better understanding of these biases will enhance our ability to reconstruct Pleistocene paleoenvironments, thereby providing valuable insights into how current climate change may impact biodiversity. This can greatly help us to anticipate the challenges we face with current climate change.

## Author Contributions

**Dorina Szalai:** conceptualization (equal), data curation (equal), formal analysis (equal), investigation (lead), methodology (lead), resources (equal), software (equal), visualization (equal), writing – original draft (equal). **Csaba Kiss:** formal analysis (equal), investigation (equal), methodology (equal), software (equal), validation (equal), visualization (equal). **Tamás Cserkész:** data curation (equal), investigation (equal), resources (equal), validation (equal), writing – review and editing (equal). **Attila Virág:** conceptualization (equal), formal analysis (equal), methodology (equal), resources (equal), software (equal), writing – original draft (equal). **Attila Németh:** conceptualization (equal), funding acquisition (lead), investigation (equal), methodology (equal), resources (equal), supervision (lead), writing – review and editing (equal).

## Funding

The authors have nothing to report.

## Conflicts of Interest

The authors declare no conflicts of interest.

## Supporting information


**Table S1:** Entire datasets on the cases utilised in the study.

## Data Availability

The authors make all the necessary data available as Supporting Information S1.
